# Questions and Controversies About Parathyroid Pathophysiology in Children With Multiple Endocrine Neoplasia Type 1

**DOI:** 10.3389/fendo.2018.00359

**Published:** 2018-07-17

**Authors:** Stephen J. Marx, Delmar M. Lourenço

**Affiliations:** ^1^Eunice Kennedy Shriver National Institute of Child Health and Human Development, National Institutes of Health, Bethesda, MD, United States; ^2^Endocrine Genetics Unit (LIM-25), Endocrinology Division, Hospital das Clínicas, University of São Paulo School of Medicine, São Paulo, Brazil; ^3^Endocrine Oncology Division, Institute of Cancer of the State of São Paulo, University of São Paulo School of Medicine, São Paulo, Brazil

**Keywords:** MEN1, MEN4, *CDKN1B*, parathyroid, genetic counseling, tumorigenesis

## Introduction

Multiple endocrine neoplasia type 1 (MEN1) is a heterogeneous disorder, with tumors among some 30 different tissue types (1). The tumors may be hormone-secreting or hormone non-secreting. The tumors are expressed mainly in adults; however, the penetrance of primary hyperparathyroidism (PHPT) in MEN1 is already around 50% by age 20 years (2). Few MEN1 series focusing on children have been described or collected (2–5). Thus, it is not possible to make well-supported assessments about many issues on this topic. In this article, we highlight and provide opinions about important issues that are unresolved or even controversial. Most issues focus on the parathyroids in MEN1. Some of these issues may have a different approach in other articles of this volume.

## Is there a hyperplastic precursor to the neoplastic parathyroid tumor in MEN1?

Knowledge about a hyperplastic precursor stage to parathyroid tumors in MEN1 could assist in planning interventions before a less responsive neoplastic mono- or oligo-clonal stage. Hyperplasia is often defined by presence of polyclonal secretory tissue (6). Advanced parathyroid tumors in adults with MEN1 always or usually represent mono- or oligo-clonal cellular proliferation that probably arises independently and randomly in each of the parathyroid glands of the same patient (7–9). Hyperplasia has not been explored in the early hyperparathyroid tissue of MEN1; parathyroid hyperplasia would be very difficult to identify therein by histology. However, a hyperplastic precursor stage has been identified before expression of gastrinoma in MEN1, making it likely that hyperplasia is also a precursor of the parathyroid tumor in MEN1 (10).

## What is the typical age of onset of parathyroid tumors and PHPT in MEN1? is there ever an onset *in utero* in MEN1?

Age of onset of PHPT in MEN1 helps guide the earliest ages to offer genetic screening. Since PHPT has a high penetrance in adolescents with MEN1, it would be of some interest to know how early parathyroid tumors and PHPT begin in MEN1. PHPT in neonatal severe PHPT always or often begins *in utero* (11). This is usually from mutation in *CASR* encoding the calcium-sensing receptor, and expressed mainly upon parathyroid cells (12). Similar early onset stage may also be considered in MEN1. Since one or two parathyroid glands seem normal even at parathyroid surgery beyond age 20 in MEN1 (13), it is likely that childhood parathyroid neoplasia in one or more glands in MEN1 begins postnatally, i.e., not *in utero*. The time interval—probably variable and multifactorial—between occurrence of the second mutational event in a tumor precursor parathyroid cell and the onset of hypercalcemia is unknown in MEN1.

No systematic screening for PHPT in MEN1 has been reported during the first years of life. Only three children younger than age of 6 years (4–5 years) have been reported with asymptomatic hypercalcemia in MEN1 (5). Thus, hypercalcemia seems to be rare in MEN1 for this age group. The usual age of diagnosis of PHPT in MEN1 is at the beginning of the third decade of life (20–25 years) (2, 9). In the largest series about children with MEN1, most (58%; 71/122) of the young cases were diagnosed with PHPT between 15 and 20 years, with progressively lower frequencies between 10–15 years (32%; 39/122), and during the first decade of life (10%; 12/122). Overall, 17% (21/122) of all children with MEN1 were considered to be symptomatic from PHPT (5).

## Does early onset of PHPT in MEN1 accompany tumor in fewer parathyroid glands? does early onset reflect an unusually aggressive course?

In a subset of 38 young cases (8–20 years) of MEN1 with PHPT, most were submitted to less than subtotal parathyroidectomy (25, 68%). Sixty seven percent of those with less than subtotal parathyroidectomy had normal calcemic status in the last follow-up before 21 years (5). These data favor, but do not prove, a preferential involvement of a lesser number of parathyroid glands at earlier ages.

One index of aggressivity of PHPT is skeletal status. It is possible that early onset of PHPT may interfere with peak of bone mass. In fact, reduced bone mineral density was observed in half of a small subset of patients younger than 30 years with MEN1 and PHPT (14, 15). Thus, a potentially more aggressive course concerning skeletal mass could occur in young patients, who had not reached the maximum peak of bone mass. This type of aggressivity relates to early age of onset and longer exposure of the growing skeleton to PHPT in MEN1. Thus, it does not prove more aggressivity of the parathyroid tumors.

## Does early onset justify operation on fewer parathyroid glands?

The parathyroid disease in MEN1, classically multiglandular, asymmetric, and asynchronous has motivated some surgeons, more recently, to perform more conservative operations, i.e., less than 4 gland subtotal parathyroidectomy. This approach is controversial. This is based on resection directed only to enlarged parathyroid glands, as identified on pre-operative radiological localization and significant drop of intra-operative PTH values (9, 16). However, these results are very preliminary, and the benefits of this conservative surgery need to be contra-balanced with higher risk of persistent PHPT and risks arising from potential for multiple parathyroid surgeries. Overall, limited parathyroidectomy (≤3 glands) in adults with MEN1 was associated with a higher rate of postoperative persistent PHPT and should not be performed routinely in children with MEN1 (17).

## What is the diagnostic value of early onset of hypercalcemia in MEN1?

MEN1 and familial hypocalciuric hypercalcemia have approximately equal prevalence. The earliest PHPT in MEN1 was reported in a child of 4 years (5). However, hypercalcemia has been rarely reported during the first decade in MEN1 with no more than 16 cases reported in the two large MEN1 series (2, 5). Thus, MEN1 becomes more relevant in the differential diagnosis of hypercalcemia after the age of 8 years. More rare causes of hypercalcemia that might be encountered after age 8 are the hyperparathyroid-jaw tumor syndrome, familial isolated hyperparathyroidism, familial hyperparathyroidism without known mutation, and nonfamilial hyperparathyroidism (11). Before age 8, almost all cases of PTH-dependent hypercalcemia are caused by familial hypocalciuric hypercalcemia (mostly from mutation in *CASR, AP2S1, or GNA11*) or by neonatal severe primary hyperparathyroidism, both of which have near 100% penetrance for hypercalcemia already in the neonate (12, 18). Thus, the age at the diagnosis of hypercalcemia is one of the important clues for diagnosis of MEN1. Importantly, different strategies of clinical management are recommended in each of these genetic disorders (11).

## What are the indications for early surgery of PHPT in MEN1?

The recent guidelines for asymptomatic PHPT suggest offering surgery to all cases below age 50 years (19). This would be controversial in MEN1, where recurrent PHPT is 50% at 12 years after seemingly successful parathyroid surgery in adults (20). We suggest reserving parathyroid surgery in children with MEN1 for any of the following indications: serum calcium more than 1 mg% (0.25 mM) over the upper normal, nephrolithiasis, nephrocalcinosis, reduced bone mineral density (Z score below −2), pathologic fracture, and failure of therapy to correct hypercalciuria with diuretics. Further controversies on potential indications, ideal timing, and extension of the surgery are discussed in other articles of this special issue. If parathyroid surgery is indicated, it is imperative in this age group to secure the longest period of normocalcemia to reach the maximum accretion of bone mass during the second and third decade of life. Importantly, permanent hypoparathyroidism should be avoided, when possible, as this condition is associated with lower quality of life (21, 22), including lower adherence to calcemic therapy for children.

## Should cinacalcet ever be given to a child with MEN1?

Cinacalcet is a calcimimetic, an allosteric agonist at the calcium-sensing receptor on the parathyroid cell. It is effective to lower PTH in primary and secondary hyperparathyroid states including PHPT of MEN1 (23–25). Its use has not been reported in children with MEN1. Importantly, there was death in one hypocalcemic 14 year-old child during a trial of Sensipar (cinacalcet) for uremic hyperparathyroidism (26) (FDA is Food and Drug Administration); the cause of this death has not been reported and trials of cinacalcet in children were suspended by the FDA. Thus, cinacalcet should be reserved for off label use in children with MEN1 only with strong indications. These mainly include symptoms of PHPT with lack of control by parathyroid surgery and parathyroid cancer. Parathyroid cancer has not been reported in a child with MEN1 (27).

## How early should mutation screening for *MEN1* be offered in an asymptomatic family member?

There is controversy about offering a genetic test at an age when the patient cannot decide maturely on its desirability. Potential benefit to the child is a major criterion (28). We feel that use of the test in planning screens for potentially morbid traits is a justification that can override the patient's incomplete understanding of the test. Since morbid prolactinoma has been reported at age 5 in MEN1, we consider this as the earliest age to offer genetic testing for MEN1 (29). However, since this is a rare expression of such early MEN1, the decision to undertake genetic testing at or around this age should be made carefully, together with the parents or guardians.

A separate topic about early testing in relation to a family member is *in vitro* fertilization and testing with tissue from a 5-cell embryo or similar specimen. This would be for use in preimplantation genetic diagnosis (30). This has not been reported, but it has been done for *MEN1* mutation (SJM personal communication).

## Is MEN4 different clinically from MEN1?

MEN4 is a MEN1-like case or family with no *MEN1* mutation but with mutation of *p27* or another cyclin-dependent protein kinase inhibitor gene. MEN4 cases show tumors typical of MEN1 (31). Skin tumors like those in MEN1 have not been reported in MEN4. Only 19 MEN4 patients have been reported with germline mutations in *p27* and seven with mutation in *p15* (*n* = 3), *p18* (*n* = 2), or *p21* (*n* = 3). Most of them represented isolated cases or very small families (31–33). Thus, there are not enough data to determine if MEN1 and MEN4 differ in phenotype. However, based on limited data, PHPT in MEN4 seems to be diagnosed later than in MEN1; mean age at the diagnosis in 17 MEN4 patients was 53 years (30, 34). Up to now, only one case younger than 20 years presenting as apparently sporadic PHPT was diagnosed as MEN4 (35).

## Do mutation positive cases of MEN1 differ from mutation negative cases?

Approximately 75–95% of MEN1 cases have a small *MEN1* deletion/insertion, or splice change, or point mutation (1, 9, 36). One percent have a large deletion of *MEN1* (37). One to two percent have mutation of *p27* or another cyclin-dependent protein kinase inhibitor (32). Those cases without identified mutation (or “mutation negative”) are older, live longer, and develop frequently no more than two main MEN1-related tumors (38). If there is a pituitary tumor in a *MEN1* mutation negative case, it is more likely to be GH-secreting than the usual PRL-secreting tumor of MEN1 (39). Overall the mutation negative cases do seem to differ in phenotype from those with identified mutation.

## What is the molecular pathway for tumorigenesis in MEN1?

Knowing the molecular pathway for tumorigenesis in MEN1 could help to determine intrinsic action mechanisms and especially what molecules in the MENIN molecular pathway should be candidates for targeted intervention. Twenty years after *MEN1* gene discovery in 1997 (40), MENIN's molecular pathway remains unproven. Most work has been directed at finding binding partners of MENIN, and some 30 partners have been reported (41). The partners studied in greatest detail have been junD and mixed lineage leukemia (MLL). Remarkably, junD and MLL were recently shown to have a small domain of sequence homology, at which each might interact with MENIN and differently from each other (42).

## Author contributions

Both authors listed have made a substantial, direct and intellectual contribution to the work, and approved it for publication.

### Conflict of interest statement

The authors declare that the research was conducted in the absence of any commercial or financial relationships that could be construed as a potential conflict of interest.

## Abbreviations

FDA: Food and Drug Administration
MEN1: multiple endocrine neoplasia type 1
PHPT: primary hyperparathyroidism.
